# A case of non-ampullary duodenal adenosquamous carcinoma with successful emergency pancreaticoduodenectomy for gastrointestinal hemorrhage

**DOI:** 10.1186/s40792-023-01749-x

**Published:** 2023-09-12

**Authors:** Arimasa Miyama, Yuko Chikaishi, Daigo Kobayashi, Kazuhiro Matsuo, Takayuki Ochi, Kenichi Nakamura, Tomoyoshi Endo, Kenji Kikuchi, Hidetoshi Katsuno, Aki Nishijima, Zenichi Morise

**Affiliations:** 1 Department of Surgery, Fujita Health University School of Medicine Okazaki Medical Center, 1 Gotanda Harisakicho, Okazaki, Aichi 444-0827 Japan; 2 Department of Diagnostic Pathology, Okazaki Medical Center, Fujita Health University School of Medicine, Okazaki, 444-0827 Japan

**Keywords:** Non-ampullary duodenal adenosquamous carcinoma, Emergency pancreaticoduodenectomy, Gastrointestinal hemorrhage

## Abstract

**Background:**

Although most duodenal carcinomas are pathological adenocarcinomas, a small number of cases have been reported of adenosquamous carcinoma, characterized by variable combinations of two malignant components: adenocarcinoma and squamous cell carcinoma. However, owing to the small number of cases of non-ampullary duodenal adenosquamous carcinoma, there have been no reported cases of emergency pancreaticoduodenectomy for gastrointestinal hemorrhage due to non-ampullary duodenal adenosquamous carcinoma.

**Case presentation:**

A 66-year-old Japanese male presented to the referring hospital with a chief complaint of abdominal pain, diarrhea, and dark urine that had persisted for 1 month. The patient was referred to our hospital because of liver dysfunction on a blood examination. Laboratory results of the blood on the day of admission showed that total and direct bilirubin levels (12.0 mg/dl and 9.6 mg/dl) were markedly increased. An endoscopic retrograde biliary drainage tube was inserted for the treatment of obstructive jaundice, and imaging studies were continuously performed. Contrast-enhanced computed tomography and endoscopy revealed an ill-defined lesion involving the second portion of the duodenum, predominantly along the medial wall, and measuring 60 mm in diameter. No metastases were observed by positron emission tomography. Pancreaticoduodenectomy was planned based on the pathological findings of poorly differentiated adenocarcinoma. However, 2 days before the scheduled surgery, the patient experienced hemorrhagic shock with melena. Owing to poor hemostasis after endoscopic treatment and poor control of hemodynamic circulation despite blood transfusion, radiological embolization and hemostasis were attempted but were incomplete. An emergency pancreaticoduodenectomy was performed after embolizing the route from the gastroduodenal artery and pseudoaneurysm area to reduce bleeding. The operation was completed using an anterior approach without Kocherization or tunneling due to the huge tumor. The operation time was 4 h and 32 min, and blood loss was 595 mL The pathological diagnosis was adenosquamous carcinoma. The postoperative course was uneventful with 17 day hospital stay and the patient is currently well, with no signs of recurrence 9 months after surgery.

**Conclusions:**

This report presents an extremely rare case of successful emergency pancreaticoduodenectomy for gastrointestinal hemorrhage caused by non-ampullary duodenal adenosquamous carcinoma.

## Background

The incidence of duodenal carcinoma has recently increased due to the widespread use and development of endoscopic examination [1, 2]. Although most duodenal carcinomas are adenocarcinomas, a small number of cases have been reported of adenosquamous carcinoma (ASC), characterized by variable combinations of two malignant components: adenocarcinoma and squamous cell carcinoma [3, 4]. Non-ampullary duodenal adenosquamous carcinoma (NADASC) is rare. Xue et al. reported only one case of NADASC in their study of 47 non-ampullary duodenal carcinomas [5]. To date, only five cases have been reported in the literature [6–10]. ASCs in other primary sites that normally have glandular epithelium, such as the pancreas, stomach, colon, rectum, breast, and prostate, are also rare and considered to be clinically more aggressive, with a worse prognosis than their adenocarcinoma counterparts [6, 7, 9]. Surgical resection is the only curative treatment for advanced duodenal carcinomas, including NADASC, and most reported patients have undergone pancreaticoduodenectomy (PD). However, owing to the small number of NADASC cases, there have been no reported cases of emergency pancreaticoduodenectomy (ePD) for gastrointestinal hemorrhage caused by NADASC. This report presents an extremely rare case of successful ePD for gastrointestinal hemorrhage caused by a NADASC.

## Case presentation

A 66-year-old Japanese male presented to the referring hospital with a chief complaint of abdominal pain, diarrhea, and dark urine that had persisted for 1 month. The patient was referred to our hospital because of liver dysfunction on a blood examination. He had a history of treatment for hypertension, diabetes mellitus, and dyslipidemia, but no history of liver dysfunction or habitual alcohol consumption.

Laboratory results of the blood on the day of admission showed that total and direct bilirubin levels (12.0 mg/dL and 9.6 mg/dL) were markedly increased. Mild elevations in the aspartate aminotransferase (56 U/L), alanine aminotransferase (50 U/L), gamma-glutamyl transpeptidase (296 U/L), alkaline phosphatase (196 U/L), and amylase levels (249 U/L) were observed. Carbohydrate antigen 19–9 was elevated at 48.9 U/mL, but the carcinoembryonic antigen level was normal at 3.6 ng/mL. Tests for hepatitis B virus surface antigen and hepatitis C virus antibodies were negative.

**Fig. 1:**
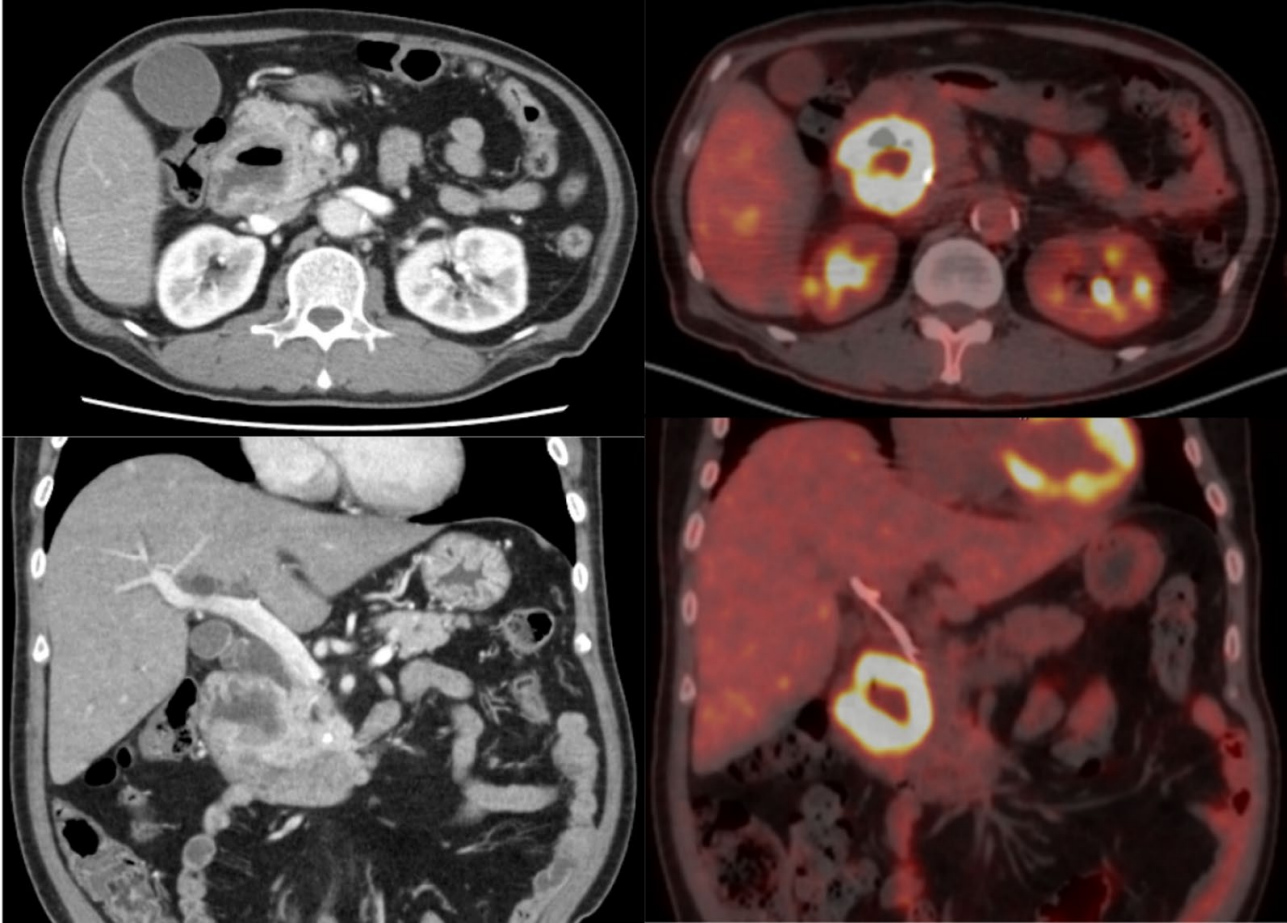
Findings of contrast-computed tomography (CT) and positron emission tomography (PET)–CT: contrast-CT revealed an ill-defined lesion involving the second portion of the duodenum, predominantly along its medial wall, measuring a diameter of 60 mm. Bulging growth into the duodenal lumen and the pancreatic head, suggesting infiltration of the lesion, was observed. In addition, associated with gallbladder enlargement, bile duct dilation due to the stenosis of the lower bile duct, and dilation of the main pancreatic duct. No metastasis was observed on PET–CT

**Fig. 2:**
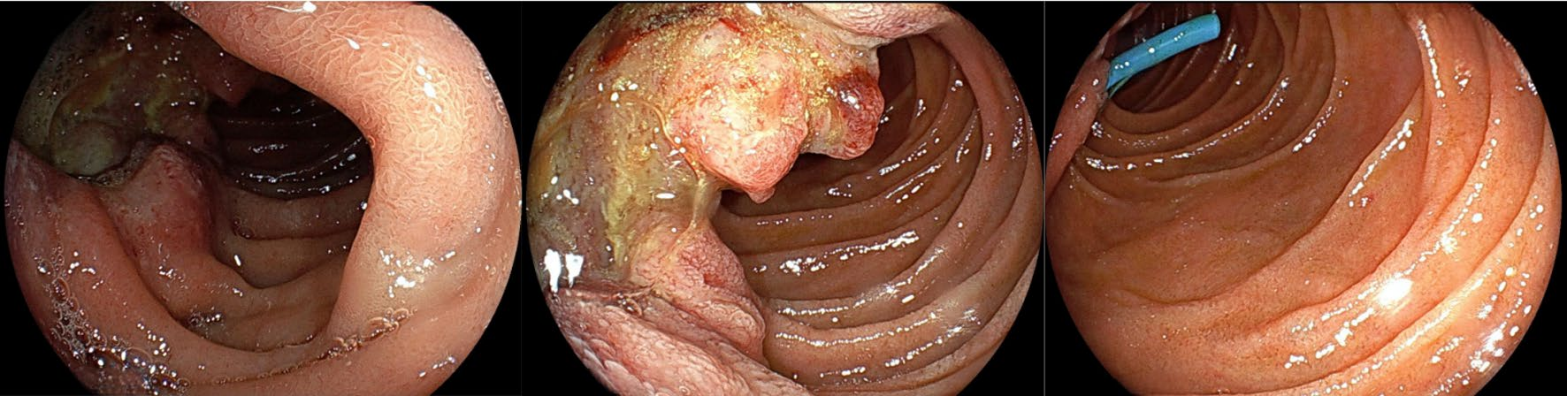
Findings of upper gastrointestinal endoscopy: the examination revealed a protuberant lesion in the second partition of the duodenum. The tumor was located separately from the duodenal ampulla

An endoscopic retrograde biliary drainage (ERBD) tube was inserted for obstructive jaundice, and endoscopic and imaging evaluations were performed continuously. Computed tomography (CT) of the abdomen showed a 60-mm lesion in the area of the 2nd portion of the duodenum and pancreatic head, associated with gallbladder enlargement, bile duct dilation due to stenosis of the lower bile duct, and dilation of the main pancreatic duct. Contrast-enhanced CT revealed that the lesion originated from the medial wall of the duodenum, with irregular wall thickening, and extended consecutively to the pancreatic head, with the invasion of the common bile duct and main pancreatic duct. The anterior superior, posterior superior, and inferior pancreaticoduodenal arteries (IPDA), forming the network between the gastroduodenal and superior mesenteric arteries (SMA), were considered the feeding arteries. Positron emission tomography (PET)-CT showed accumulation (SUV: 13.3) at the lesion; however, there were no abnormal accumulation findings at other sites (Fig. 1). Upper gastrointestinal endoscopy revealed a protuberant lesion with deep ulceration in the second portion of duodenum. The lesion was located orally and was separated from the duodenal ampulla (Fig. 2). Pathological result of the endoscopic biopsy specimen was poorly differentiated adenocarcinoma.

**Fig. 3:**
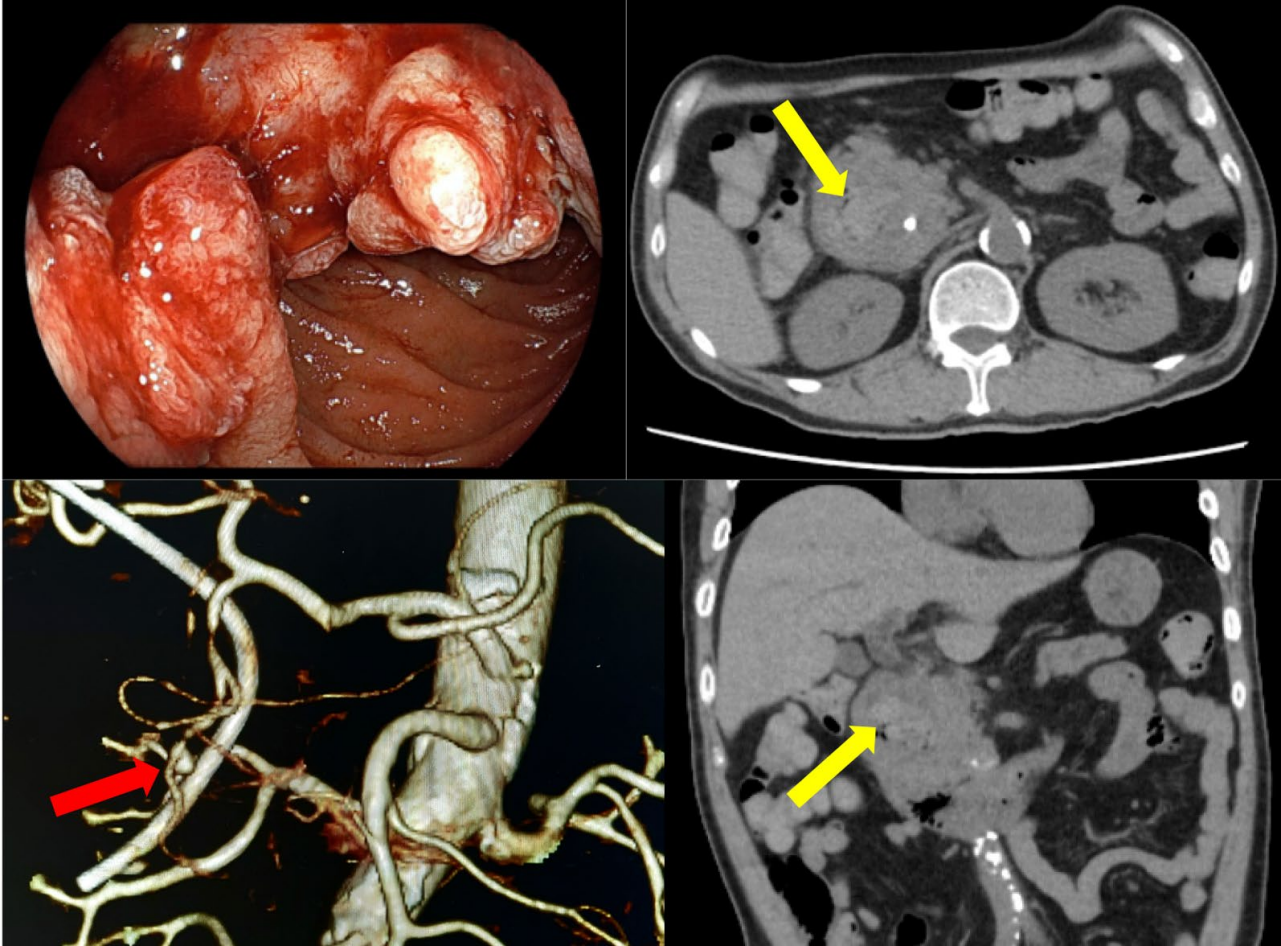
Findings of Emergency Endoscopy and Computed Tomography (CT), 3D-CT Angio: active hemorrhage from the duodenal tumor was observed on emergency endoscopy. A high-density region (yellow arrow) in the lumen of the lesion, suggesting an intraluminal hemorrhage and pseudoaneurysm formation (red arrow) in the gastroduodenal artery (GDA) branch (anterior superior pancreaticoduodenal artery) on CT and 3D-CT

Although elective PD was planned, the patient experienced hemorrhagic shock with melena on 2 days before the scheduled surgery. Emergency gastrointestinal endoscopy was performed and found out the bleeding origin, the deep ulceration area in the duodenal tumor (Fig. 3). However, the endoscopic attempt of achieving hemostasis was failed. His hemoglobin level was decreased to 8.1 from 11.2 g/dl 2 days earlier. With the arterial pressure recovered to 100 mmHg by blood transfusion, the patient underwent CT with contrast. The CT showed a high-density region in the ulceration space of the tumor, suggesting intraluminal hemorrhage and pseudoaneurysm formation in the gastroduodenal artery (GDA) branch (anterior superior pancreaticoduodenal artery) at the bottom of the ulceration (Fig. 3).

**Fig. 4:**
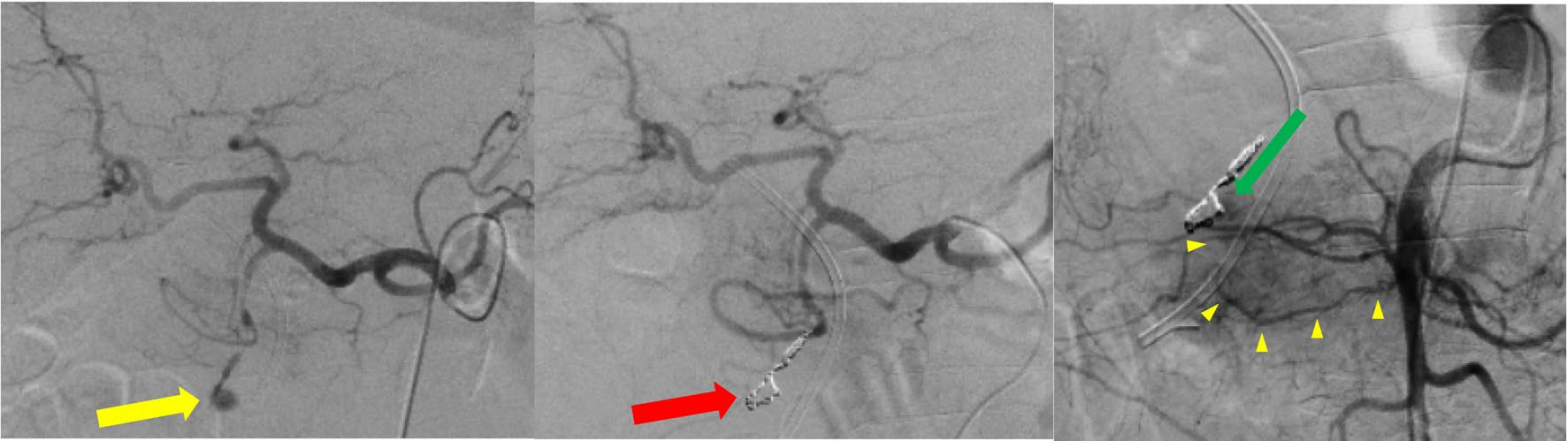
Findings of emergency angiography: a radiological embolization and hemostasis from the gastroduodenal artery (GDA) for the treatment of the pseudoaneurysm (yellow arrow) on the GDA-inferior pancreaticoduodenal artery (IPDA, yellow arrow heads) arcade was tried (red arrow). However, since the GDA–IPDA arcade with blood supplies from both celiac and superior mesenteric arteries was entrapped in the intrapancreatic ulcer bed at the base of the tumor and disrupted, hemostasis was uncompleted (green arrow)

Due to the failure of hemostasis by endoscopic intervention and poor control of hemodynamic circulation despite blood transfusion, radiological embolization and hemostasis on angiography were attempted (Fig. 4). Since the IPDA branch from SMA was thin and irregular with suspicious invasion of the cancer (Fig. 4, yellow arrow heads), the cannulation to the artery was failed. Although a catheter was inserted to the aneurysm and tried to reach the distal lumen of IPDA, the guide-wire strayed out to the duodenal lumen and the distal lumen of the arterial arcade could not be reached. His systolic arterial pressure temporarily decreased to 50 mmHg during the IVR. The route only from the GDA and pseudoaneurysm areas was embolized to reduce bleeding, and ePD was continuously performed with a massive blood transfusion for maintaining the circulation. At the beginning of the surgery, his hemoglobin level was 6.6 g/dl even after 10-unit transfusion of packed red blood cells.

During surgery, there were no ascites or peritoneal seeding findings, and a > 80 mm tumor was found in the pancreaticoduodenal region. The intestinal tract, mainly from the duodenum to the jejunum, was tense, with blood stored in the lumen, although there was no intraabdominal bleeding. The first part of the jejunum was transected to close the tract and reduce bleeding. It was dissected from the transverse mesocolon toward the ligament of Treitz. The right side of the transverse mesocolon was incised, and the first part of the jejunum was pulled out from the ligament of Treitz to the right. The right side of the superior mesenteric artery was approached to control bleeding by blocking the blood supply from the SMA into the tumor. However, as the mesentery on the right side of the SMA was tightly constricted, only the caudal side could be dissected. The gastrocolic ligament was opened, the stomach was resected at the pylorus, and the hepatoduodenal ligament was dissected by transecting the common hepatic duct and GDA. Since the tumor was so large that the usual tunneling procedure between the pancreas and superior mesenteric vein (SMV) was difficult, the pancreas was gradually divided from the caudal side without a tunneling procedure, and the SMV anterior surface was exposed. Thereafter, the right side of the SMV/SMA/plexus was dissected from the uncinate process of the pancreas in an anterior-to-posterior direction. Finally, the pancreatic head and duodenum containing the tumor were dissected from the retroperitoneum and removed. The operation was completed using an anterior approach without Kocherization or tunneling. The operation time was 4 h and 32 min, and blood loss was 595 mL. The hemoglobin level was elevated to 9.8 g/dl after the surgery with total of 26-unit red blood cells transfusion.

**Fig. 5:**
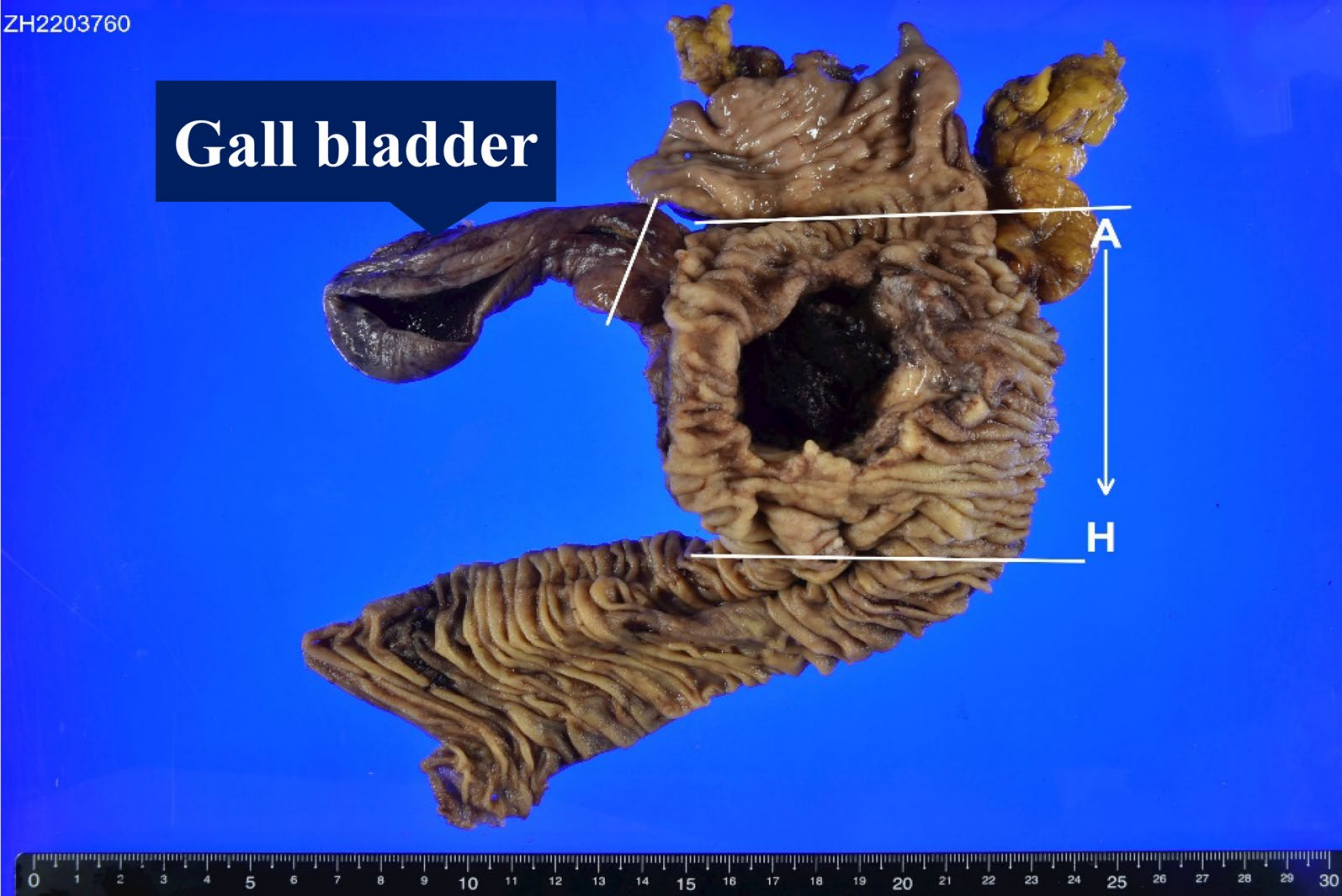
Findings of the resection specimen: the resection specimen showed a type 3 tumor 8 cm in size that invaded from the duodenum to the pancreatic head and formed a deep ulcer with vascular embolization coils at the base of the ulcer

**Fig. 6:**
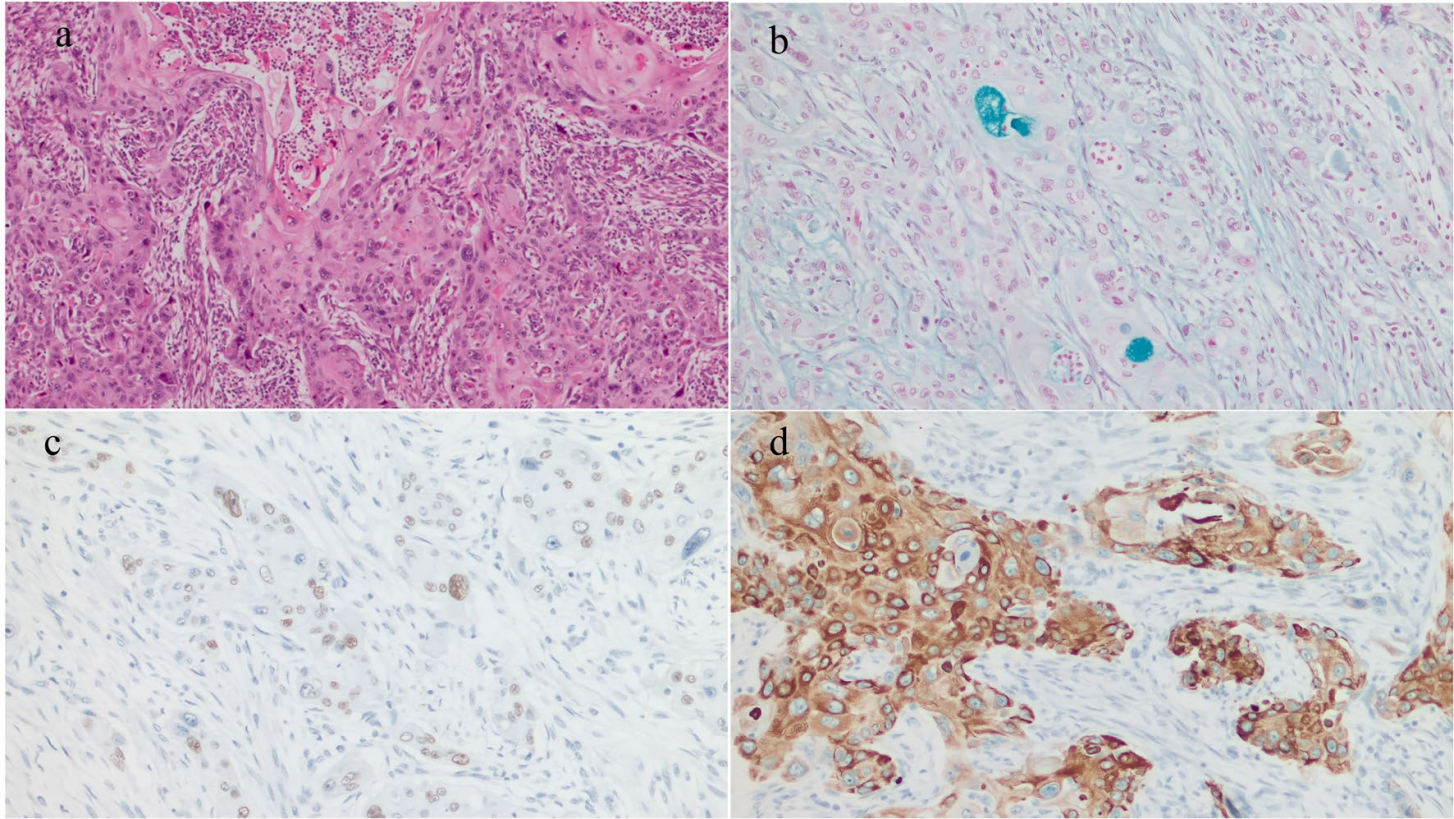
Findings of pathological examination: pathological examination of the tumor area showed carcinoma cells arranged in nests and trabecular patterns, with scattered keratinization within the nests. The present case showed a small number of glandular ducts that were positive for hematoxylin–eosin (**a**) and alcian blue staining (**b**), and the majority of tumor cells were p40 (**c**) and cytokeratin5/6 (CK5/6) (**d**) positive. The pathologic diagnosis was adenosquamous carcinoma

The resected specimen showed an 8-cm type 3 tumor that invaded from the duodenum to the pancreatic head and formed a deep ulcer with vascular embolization coils at the base of the ulcer (Fig. 5). Pathological examination of the tumor revealed carcinoma cells arranged in nests and a trabecular pattern with scattered keratinization within the nests. The majority of the carcinomas were composed of squamous cell carcinoma components, but a few areas of suspected glandular differentiation were observed (Fig. 6a). A small number of glandular ducts positive for Alcian blue staining (Fig. 6b) were observed in the specimen, and the majority of tumor cells were positive for p40 (Fig. 6c) and cytokeratin5/6 (CK5/6) (Fig. 6d). The tumor gland ducts were negative for mucin (MUC)2, MUC5AC, MUC6, and caudal-related homeobox transcription factor-2 (CDX-2). The final pathological diagnosis of ASC was made. In addition, three lymph node metastases were observed on the anterior and dorsal surfaces of the pancreatic head.

The patient’s postoperative course was uneventful, and he was discharged on the 17th postoperative day. On postoperative day 58, S-1 (tegafur/gimeracil/oteracil potassium) plus oxaliplatin therapy (SOX) was initiated as adjuvant chemotherapy. The patient is currently well, with no signs of recurrence 9 months after surgery.

## Discussion

The pathogenesis and natural history of NADASC are unknown owing to their rarity. The consensus regarding the treatment of these infrequent carcinomas is surgical resection of the tumor with negative margins, irrespective of histology. Furthermore, when duodenal carcinoma is large and deeply invades the pancreatic head, it may cause bleeding due to invasion of the pancreatic head arcade artery, as in the present case. Arteries in this region receive blood flow from both the celiac artery and the SMA, and hemostasis by interventional radiology can be difficult and requires surgical intervention. There are few reports in the literature on ePD for duodenal carcinoma. Only three reports have described ePD as a lifesaving procedure for gastrointestinal hemorrhage from duodenal carcinoma in detail [11–13] (Table 1). Of these reported cases, two were adenocarcinomas and one was a carcinoma without a detailed description. One of these was a non-ampullary duodenal adenocarcinoma. Standop et al. reported that the patient experienced 63 days of postoperative hospitalization with delayed gastric emptying and acute renal failure but survived [11]. Ye et al. reported that the patient still suffered from persistent gastrointestinal bleeding after surgery and died 1 month postoperatively due to an intraabdominal infection [12]. Emiloju et al. reported that the patient died 90 days after surgery. She was not a candidate for any adjuvant therapy with a performance status of 3 and died of the disease owing to its aggressive nature and poor prognosis [13]. Fortunately, the postoperative course was uneventful, and the patient is well without any signs of recurrence in the present case. The reported outcomes of ePD for duodenal carcinoma are unfavorable, since the patients had far-advanced diseases. The application of ePD in patients with distant metastases or other incurable factors, who are out of usual surgical indication as cancer treatment, should be carefully considered. However, ePD in the present case resulted in good mid-term outcomes, though he had no incurable factors but advanced disease. For lifesaving purposes, this procedure can be applied to patients without massive metastases that limit short-term survival, resulting in good mid-term outcomes, even when they have the diseases out of surgical indication oncologically.

**Table 1:** Reports of emergency PD for gastrointestinal hemorrhage due to duodenal carcinoma

Year	Author [reference number]	Age	M/F	Size (mm)	Location	Pathologic diagnosis	Complication	Length of stay, (days)
2010	Standop et al. [11]	74	F	NA	Papillary	NA	Delayed gastric emptying, acute renal failure	63
2020	Emiloju et al. [13]	60	F	90	NA	ADENO	Abdominal dehiscence	90 (hospital-death of the carcinoma)
2022	Ye et al. [12]	82	M	100	Bulb	ADENO	Melena, atelectasis, ileus, intra-abdominal infection	30 (hospital-death)
2023	Present case	66	M	80	2nd	ADENOSQUAMOUS	No complications	17

*M* male, *F* female, *NA* not available

Only five cases of primary duodenal ASC of the non-ampulla of Vater have been reported in the English literature [6–10] (Table 2). NADASCs appear to affect both males and females, but the reported ratio of males to females is 5:1. The most common clinical symptoms are abdominal pain and melena. In the present case, abdominal pain, diarrhea, and dark urine (obstructive jaundice) were the primary symptoms. Although the present case was a non-ampullary duodenal carcinoma, direct tumor invasion of the common bile duct in the pancreatic head caused obstructive jaundice. Surgical resection (PD) of the tumor is the predominant treatment modality; however, the prognosis is unknown owing to the short follow-up period in the reported cases. In our case with following adjuvant chemotherapy after the ePD, there was no evidence of local recurrence or distant metastasis on CT findings 9 months after surgery.

**Table 2:** Case of adenosquamous carcinoma of non-ampulla of Vater reported in literature

Year	Author [reference number]	Age	M/F	Symptom	Size (mm)	NAC	Surgery	ePD	LN metastasis	Location	Postoperative adjuvant chemotherapy	Postoperative distant metastasis	Prognosis (months)
2011	Hsueh et al. [6]	67	M	Dyspnea on exertion, abdominal pain, melena	81	(−)	PD	(−)	(+)	3rd	NA	NA	NA
2016	Daga et al. [7]	78	M	Melena	30	(−)	PD	(−)	(−)	2nd	NA	(−)	3 alive
2016	Takayoshi et al. [8]	76	M	Epigastric distress	50	(−)	PD	(−)	NA	2nd	1st FOLFOX 2nd CPT-11,Cmab	(+)	26 dead
2017	Hammami et al. [9]	64	F	Abdominal discomfort, vomiting, abdominal pain, altered mental status	NA	(−)	Gastrojejunostomy	(−)	(+)	3rd	(-)	(+)	NA
2018	Sim et al. [10]	59	M	Melena	40	(−)	Duodenal segmental resection with gastrojejunostomy	(−)	NA	Bulb	NA	(-)	3 alive
2023	Present case	66	M	Abdominal pain, diarrhea, dark urine	80	(−)	PD	(+)	(+)	2nd	SOX	(-)	8 alive

*M* male, *F* female, *NAC* neoadjuvant chemotherapy, *PD* pancreaticoduodenectomy, *ePD* emergency pancreaticoduodenectomy, *LN* lymph node, *FOLFOX* folinic acid/5-fluorouracil and oxaliplatin, *CPT-11* camptothecin-11, *Cmab* cetuximab, *SOX* tegafur/gimeracil/oteracil potassium and oxaliplatin, *NA* not available

Morbidity and mortality have been reported to increase with the ePD [11, 14]. Accordingly, ePD is only performed as a lifesaving procedure in exceptional situations such as complex pancreatic injuries, hemorrhage from ulceration or tumors that cannot be controlled conservatively or with endoscopic/radiological interventions, gross duodenal perforations, or severe infections. There are several reported cases of massive duodenal bleeding. Bleeding caused by metastatic cancers invading duodenum from outside the pancreaticoduodenal area were often handled with radiological intervention [15–17]. Bleedings from those tumors without deep invasion into the pancreatic head of rich arterial network may be handled radiologically. On the other hand, the tumor invaded deeply into pancreatic head often underwent ePD [18]. In the present case, bilateral blood supplies (from GDA and IPDA) and also thin, irregular, deviated arteries with suspicious tumor involvement made radiological hemostasis difficult to be completed. Large tumors with high vascularity deeply invaded into pancreatic head often have multiple blood supplies by tumor vessels. Radiological intervention often failed to control bleeding for those fragile tumors with central necrosis (ulceration). The technical challenges of ePD are frequently compounded by underlying malignancies, grave general conditions, septicemia, or coagulation disorders. For the patients in poor general condition, radiological hemostasis followed by elective PD is recommended. However, as in the present case, when hemostasis of a gastrointestinal hemorrhage is difficult with endoscopic and radiological interventions, ePD was the only option.

For the purposes of early bleeding control and handling the fragile tumor, the PD procedure in this case of emergency was modified from the planned one. Although Kocherization and tunneling between the pancreas and the SMV anterior surface are performed in the early stage of our usual PD procedure and had been planned also in the scheduled surgery, both were not done in this case. Huge tumor was the obstacle for these procedures and could have taken time before achieving bleeding control. Since it is necessary to control bleeding in the early stage of the operation, division of jejunum was first performed and the right side of SMA was approached to control the blood flow from IPDA. The pancreas was gradually divided from the caudal edge, exposing SMV anterior surface. The right side of the SMV/SMA/plexus was dissected from the uncinate process in an anterior–caudal to posterior–cranial direction. The operation was completed in a totally anterior approach without touching the tumor-containing duodenum and pancreas head until the final step of specimen removal by retroperitoneal dissection.

In summary, we present an extremely rare case of gastrointestinal hemorrhage due to NADASC that was successfully treated with ePD via an anterior (caudal–ventral) approach without Kocherization or tunneling in front of the SMV due to a large tumor with bleeding. Owing to the rarity of ASC in the duodenum, very limited information exists in the literature regarding radiologic findings, clinical features, and ideal management strategies. Further accumulation of cases and investigations of these uncommon carcinomas are needed.

## Conclusion

Here, we report an extremely rare case of gastrointestinal hemorrhage due to NADASC, which was successfully treated with ePD.

## Data Availability

All data generated or analyzed during this study are included in this published article.

## Abbreviations

ASC: Adenosquamous carcinoma
SCC: Squamous cell carcinoma
NADASC: Non-ampullary duodenal adenosquamous carcinoma
PD: Pancreaticoduodenectomy
ePD: Emergency pancreaticoduodenectomy
ERBD: Endoscopic retrograde biliary drainage
CT: Computed tomography
PET: Positron emission tomography
GDA: Gastroduodenal artery
IPDA: Inferior pancreaticoduodenal artery
SMA: Superior mesenteric artery
SMV: Superior mesenteric vein
CK5/6: Cytokeratin5/6
MUC: Mucin
CDX-2: Caudal-related homeobox transcription factor
SOX: S-1 (tegafur/gimeracil/oteracil potassium) plus oxaliplatin
